# Reaction Thermodynamic and Kinetics for Esterification of 1-Methoxy-2-Propanol and Acetic Acid over Ion-Exchange Resin

**DOI:** 10.3390/molecules29194709

**Published:** 2024-10-04

**Authors:** Xinyu Liu, Shu Wang, Mingxia Wang, Lifang Chen, Zhiwen Qi

**Affiliations:** State Key Laboratory of Chemical Engineering, School of Chemical Engineering, East China University of Science and Technology, 130 Meilong Road, Shanghai 200237, China; liuxy_42174@163.com (X.L.); y30230207@mail.ecust.edu.cn (S.W.); y30230119@mail.ecust.edu.cn (M.W.)

**Keywords:** 1-methoxy-2-propyl acetate (PMA), ion-exchange resin, esterification, thermodynamic, kinetic models

## Abstract

The esterification of 1-methoxy-2-propanol (PM) and acetic acid (AA) is an important reaction for the production of 1-methoxy-2-propyl acetate (PMA). Herein, we used the macroporous ion-exchange resin Amberlyst-35 as a catalyst to explore the effects of reaction conditions on the reaction rate and equilibrium yield of PMA. Under the optimized conditions of a reaction temperature of 353 K, using the initial reactant PM/AA with a molar ratio of 1:3, and a catalyst loading of 10 wt%, the PMA equilibrium yield reached 78%, which is the highest equilibrium yield so far. The reaction equilibrium constants and activity coefficients were estimated to obtain reaction thermodynamic properties, indicating the exothermicity of the reaction. Furthermore, pseudo-homogeneous (PH), Eley–Rideal (ER), and Langmuir–Hinshelwood–Hougen–Watson (LHHW) kinetic models were fitted based on experimental reaction kinetic data. The results demonstrate that the LHHW model is the most consistent with experimental data, indicating a surface reaction-controlled process and exhibiting an apparent activation energy of 62.0 ± 0.2 kJ/mol. This work represents a valuable example of calculating reaction thermodynamics and kinetics, which are particularly essential for promising industrial reactor designs.

## 1. Introduction

Methoxy propyl acetate, also known as 1-methoxy-2-propyl acetate (PMA), is widely used in paints, inks, dyes, cleaning agents, and photoresistors [1,2]. Moreover, its high solvency, thermal stability, and low toxicity have led to it gradually replacing ethylene glycol-based products [3,4,5] that have toxic effects on human metabolism [6,7,8,9]. Currently, ester exchange reactions and direct esterification are the main methods considered for the synthesis of PMA. The catalyst for ester exchange between 1-methoxy-2-propanol (PM) and methyl ethanoate [1] (or ethyl ethanoate [10]) is sodium methoxide [11], which is difficult to separate and highly susceptible to decompose in water, resulting in a reduction in catalytic activity. The direct esterification of PM with acetic acid (AA) is industrially catalyzed by sulfuric acid, which has gradually been ruled out owing to its environmental unfriendliness and corrosiveness to equipment. Thus, some environmentally-friendly catalysts, such as 4-methylbenzenesulfonic acid, titanium sulfate, and cation exchange resins, have been developed for PMA synthesis [3,12,13,14]. In addition, direct esterification of PMA synthesis is reversible and limited by a thermodynamic equilibrium [12,14,15,16,17,18,19,20,21]. Oh et al. indicated that, in a batch reactor, the maximum conversion of PM is only 46% due to the limitation of the chemical equilibrium [13].

In comparison with homogeneous catalysts, solid acid catalysts offer a number of advantages, including low corrosiveness and facile separation and, therefore, are excellent alternatives to traditional liquid acid catalysts [22,23,24]. Currently, solid acids are widely used in a variety of chemical reactions, including esterification [25,26,27], alkane isomerization [28,29], aldol condensation [30,31,32], hydrogenation [33], and formylation [34,35]. For direct esterification to synthesize PMA, Huang et al. examined the solid acid SO_4_^2−^/TiO_2_ with a pseudo-homogeneous kinetic model [3], obtaining an apparent activation energy (*Ea*_+_) of 65.7 kJ/mol. Gadekar-Shinde et al. utilized the ion-exchange resin Amberlyst-15 as a catalyst [12] and developed a concentration-based pseudo-homogeneous kinetic model with an *Ea*_+_ value of 66.5 kJ/mol. Wang et al. employed the ion-exchange resin NKC-9 as a catalyst to obtain a maximum PM conversion of 46% in a batch reaction [14], and the *Ea*_+_ was determined to be 60.5 kJ/mol based on a second-order reaction kinetic model. However, these studies tend to focus on pseudo-homogeneous (PH) kinetic models, which are unable to reveal the real reaction mechanisms related to the surface adsorption and reaction.

The Langmuir–Hinshelwood–Hougen–Watson (LHHW) and Eley–Rideal (ER) models have been extensively employed in kinetic studies of heterogeneous catalytic systems. The LHHW model describes the surface reaction between adsorbed reaction species and has been successfully employed to predict numerous reactions catalyzed by heterogeneous acid catalysts. As evidenced by previous reports, reaction kinetic data, such as esterification of benzoic acid with ethanol [36], oligomerization of 1-decene [37], liquid-phase hydrogenation of 1-indanone [38], esterification of lactic acid with ethanol [39], synthesis of tert-butyl methyl ether [40], and catalytic hydrogenation of d-lactose to lactitol [41], are well-fitted to the LHHW model. Conversely, the ER model postulates the potential for a reaction to occur between an adsorbed reactant molecule and another reactant molecule in the bulk phase. Catalytic reactions, such as cyclohexene hydration [42], the esterification of acetic acid with butanol [43], the dehydration of 1-octanol to di-n-octyl ether [44], and the oximation of cyclohexanone [45], are illustrated to follow the ER mechanism. It is noted that the model for the reaction catalyzed by a heterogeneous catalyst is rather ambiguous from case to case and should be dependent on the catalyst and reactants involved [42].

At present, the reaction kinetics for the esterification of PM with AA using a developed heterogeneous kinetic model is lacking. Herein, ion-exchange resin Amberlyst-35 was used as the catalyst, and kinetic data were obtained through the esterification of pure AA with PM in a stirred batch reactor. The effects of operating parameters, including temperature, reactant ratio, and catalyst loading, were investigated. Subsequently, this work evaluated reaction thermodynamic properties and identified several kinetic models to describe a wide range of operating conditions. 

## 2. Results and Discussion

### 2.1. Effect of Mass Transfer

**Figure 1:**
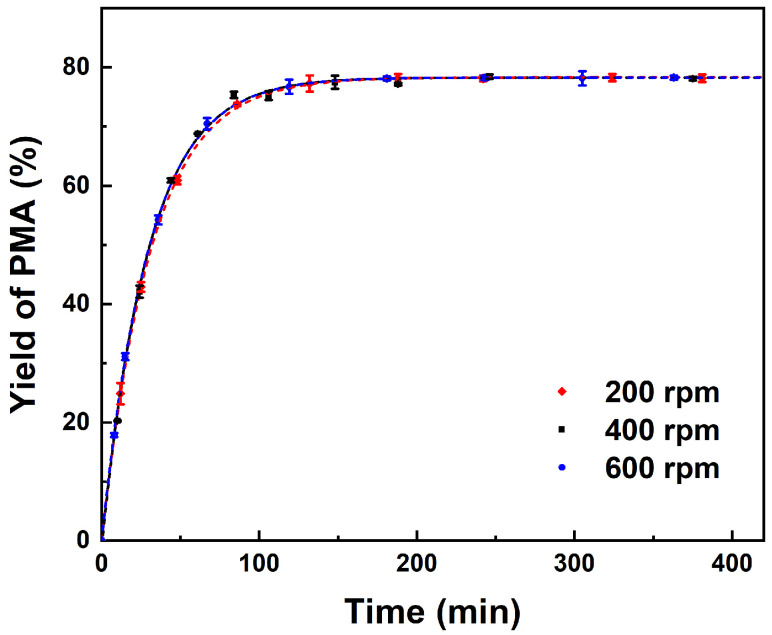
Effect of stirring speed on yield of PMA. Reaction conditions: PM:AA = 1:3 (molar ratio) and 10 wt% catalyst loading of total reactant mass at 363 K.

As indicated, the external and internal mass transfer limitations of resin catalysts in catalytic esterification reactions can be ruled out through varying stirring speeds and particle sizes of catalysts [3,13,14]. In order to avoid mass transfer influencing the reaction rate, the effect of stirring speed on PMA yield was carried out at a relatively high reaction temperature of 363 K [44]. As presented in Figure 1, it can be observed that varying stirring speeds within a range of 200 to 600 rpm has no significant impact on the yield of PMA, indicating that the influence of external diffusion can be disregarded. Nevertheless, elevated rotational speeds may result in increased wear between the rotor and catalyst particles, which is detrimental to the recovery and reuse of the catalyst. Consequently, a stirring speed of 300 rpm can be established as the optimal setting for subsequent reaction processes.

The particle diameter of the catalyst has a direct impact on internal diffusion. Previous studies have employed the method of varying catalyst particle diameters to investigate the effects of internal diffusion [13,14,46,47,48]. The particle size of the cation exchange resin Amberlyst-35 is not homogeneous, making it difficult to study the effects of internal diffusion by particle size screening. In theory, the Weisz–Prater number (*C*_wp_) can be employed to assess the influence of internal diffusion on mass transfer in a reaction system catalyzed by a cation exchange resin [49]. When *C*_wp_ is much less than 1, the effect of internal diffusion on the mass transfer resistance of the reaction is negligible. *C*_wp_ is calculated by Equation (1),

(1) $$C_{\mathrm{wp}}=\frac{-r_{\mathrm{obs}}\rho_{c}R_{c}^{2}}{C_{s}D_{e}}$$

where −*r*_obs_ represents the apparent reaction rate using an initial reaction rate to minimize the influence of external factors; *C*_s_ is the concentration of reactants on the catalyst surface, assumed to be equal to that of the liquid phase due to negligible external diffusion effects; *D*_e_ represents the effective diffusion coefficient; *ρ*_c_ is the density of catalyst; and *R*_c_ is the radius of catalyst particles (according to the report of Pera-Titus et al. [50], *ρ*_c_ is 1.542 g/cm^3^ and *R*_c_ is 0.016 cm). *D*_e_ is calculated by Equation (2),

(2) $$D_{e}=\frac{\varepsilon_{c}D_{A}}{\tau }$$

where *ε*_c_ and *τ* represent the porosity and tortuosity factors of catalyst particles (where *ε*_c_ is assumed to be 0.35 and *τ* is 1/*ε*_c_), respectively, and *D*_A_ is the infinite dilution diffusion coefficient. *D*_A_ can be calculated using the Wilke–Chang empirical correlation by Equation (3),

(3) $$D_{A}=\frac{7.4\times 10^{-8}{\left(\Phi_{2}M_{2}\right)}^{0.5}T}{\mu_{2}V_{1}^{0.6}}$$

where *Φ*_2_ is the association factor of PM, *M*_2_ is the molar mass of PM, *μ*_2_ is the viscosity of PM, and *V*_1_ is the molar volume of AA at a normal boiling point. 

Table 1 exhibits the calculated results of the Weisz–Prater number at different temperatures. Within the temperature range of 333.15 to 363.15 K, the *C*_wp_ values are significantly smaller than 1, indicating that the influence of internal diffusion can be neglected.

### 2.2. The Effect of Reaction Conditions

**Figure 2:**
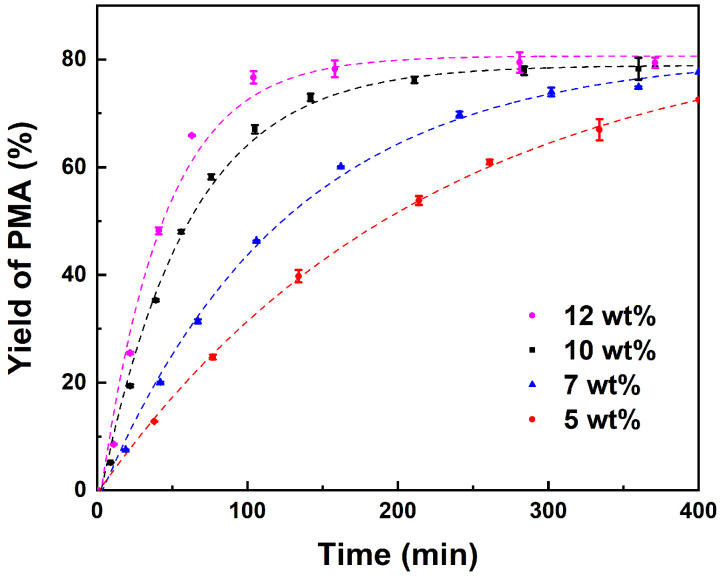
Effect of catalyst loading on yield of PMA (wt% of total reactant mass). Reaction conditions: PM:AA = 1:3 (molar ratio) at 353 K.

The impacts of various reaction parameters, including catalyst loading, temperature, and reactant molar ratio on the esterification of PM and AA were investigated. The effect of the amount of catalyst on the PMA yield was studied by varying catalyst loading from 5 to 12 wt% while all other reaction parameters were kept identical. The results, as illustrated in Figure 2, demonstrate that when catalyst loading is below 10 wt%, an increase in the catalyst dosage leads to an acceleration in the reaction rate. The maximum equilibrium yield of PMA is 78% when catalyst loading is 10 wt%. This indicates that the catalyst amount exerts an influence on the reaction rate but not on the reaction equilibrium, which is consistent with previous report [51].

However, only a slight increase in the PMA yield is observed when the catalyst loading is further increased from 10 to 12%, which is probably caused by the existence of mass transfer resistance when excess catalyst is used under the same reaction conditions [42,52]. Furthermore, the utilization of an excess of catalyst increases the cost of the reaction system. Consequently, a catalyst loading of 10 wt% is deemed to be the optimal catalyst amount for the esterification reaction between AA and PM.

**Figure 3:**
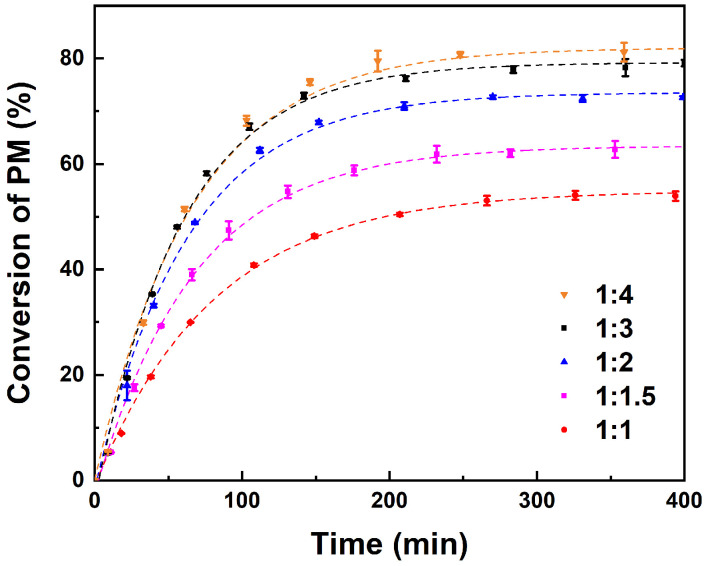
Effect of initial molar ratio of PM to AA. Reaction conditions: 10 wt% catalyst loading of the total reactant mass at 353 K.

The effect of the initial molar ratio of PM to AA on the reaction was studied by varying the molar ratios of PM to AA from 1:1 to 1:4, as shown in Figure 3, with a catalyst loading of 10 wt% and reaction temperature of 353 K. The results revealed that the conversion of PM significantly improved as the initial molar ratio of PM to AA increased from 1:1 to 1:3. Similar results have been observed in previous studies of esterification of benzyl acetate [47]. Nevertheless, as the initial molar ratio of PM to AA continued to increase to 1:4, the increased trend of PM conversion became slower. This is due to the fact that, within a certain range, an increase in the amount of AA results in an acceleration of collision frequency between reactant molecules, thereby increasing the reaction rate. Furthermore, AA also acted as a solvent to dilute the concentration of PM, which in turn results in a decrease in PM conversion. In consideration of subsequent separation issues, an excessively high concentration of AA would render separation more challenging, and consequently, the optimal initial molar ratio of PM to AA is set at 1:3.

**Figure 4:**
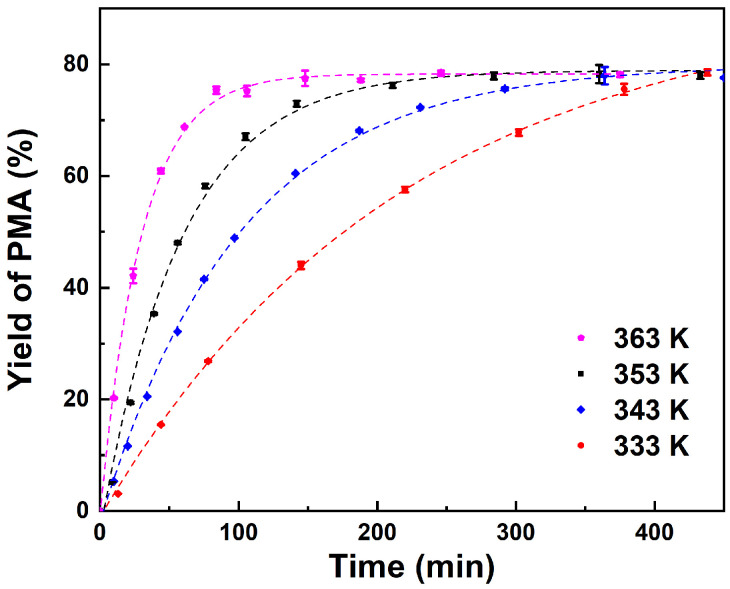
Effect of temperature on yield of PMA. Reaction conditions: PM:AA = 1:3 (molar ratio), 10 wt% catalyst loading.

In order to investigate the effect of temperature on the reaction, the molar ratio of PM to AA was fixed to 1:3 and the catalyst loading was fixed to 10 wt%. Figure 4 illustrates the relationship between reaction rate and temperature within the studied temperature range. As the reaction temperature increases from 333 to 363 K, the reaction rate accelerates, while the equilibrium yield of PMA remains relatively constant at different reaction temperatures. The experimental results indicated that the temperature had a more significant impact on the initial reaction rate than on the final reaction rate, which is consistent with previous reports [13]. An increase in temperature facilitates the free movement of molecules, thereby enhancing the frequency of collisions between reactant molecules, which in turn leads to an increase in the reaction rate [52]. Nevertheless, an increase in temperature also results in elevated energy consumption and safety concerns, and 353 K is identified as the optimal reaction temperature.

In order to evaluate the catalytic performance of Amberlyst-35 for direct synthesis of PMA, the esterification of PM and AA based on various solid catalysts are presented in Table 2. Solid acid SO_4_^2−^/TiO_2_ prepared using impregnation achieved a PMA yield of 73% under conditions of 383.15 K, 10 wt% catalyst loading, and an initial molar ratio of 1:3 [3]. As well, the NKC-9 cation exchange resin used as the catalyst exhibited a PMA yield of only 46% with 10 wt% catalyst loading and an initial molar ratio of 1:1 at 353.15 K [14]. In addition, a high PMA yield (78%) was obtained using Amberlyst-15 at 353.15 K with 10 wt% catalyst loading and an initial molar ratio of 1:3 [12]. Herein, Amberlyst-35 is comparable in catalytic performance under similar reaction conditions. This could be attributed to its higher acid capacity and larger pore size, which effectively promote reactant diffusion and provide sufficient active sites, thereby enhancing the efficiency of esterification reactions [53].

**Figure 5:**
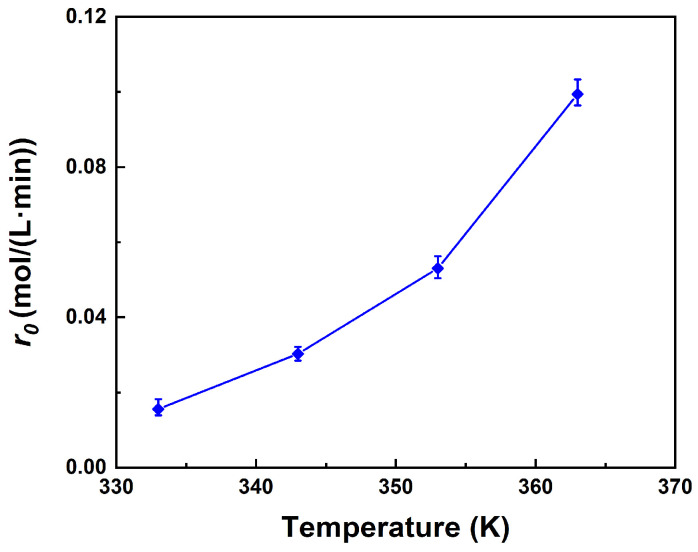
Effect of reaction temperature on the initial reaction rate. Reaction conditions: PM:AA = 1:3 (molar ratio), 10 wt% catalyst loading.

Figure 5 illustrates that the initial reaction rate that exhibits exponential growth with temperature. The calculation of the initial reaction rate is represented by Equation (4).

(4) $$r_{0}={\left(\frac{dC_{\mathrm{PM}}}{dt}\right)}_{t=0}$$

For every 10-degree increase in temperature, the initial reaction rate doubles. The substantial impact of temperature on reaction rate suggests that the reaction is regulated by either internal diffusion or surface reaction. The result has demonstrated that the esterification reaction between PM and AA is not limited by internal diffusion, as shown in Figure 1. Consequently, the surface reaction is considered to be the limiting step of the esterification reaction.

### 2.3. Chemical Reaction Thermodynamic Equilibrium

The PM-AA-PMA-H_2_O system exhibits a relatively high level of non-ideality [12,14]. In general, the non-ideality of the liquid phase mixture necessitates the use of an activity-based model. In accordance with Equation (5), the activity *α_i_* of component *i* is proportional to its mole fraction *x_i_*:

(5) $$\alpha_{i}=\gamma_{i}x_{i}$$

where *γ_i_* represents the activity coefficient of component *i*. The activity coefficient approach is applicable to liquid mixtures based on a conductor-like screening model for real solvents (COSMO-RS) model. This method enables the prediction of interaction energies and activity coefficients in complex liquid systems without the need for experimental data. The COSMO-RS model is capable of calculating the chemical potential of any solute in any pure or mixed solvent, thereby enabling the prediction of thermodynamic properties, such as activity coefficients and solubilities. 

The activity coefficient is calculated by Equation (6).

(6) $$\ln\;\gamma_{i}=(\mu_{i}^{\mathrm{sol}}-\mu_{i}^{p})/RT$$

where *μ_i_*^sol^ represents the chemical potential of solute *i* in the solvent, and *μ_i_*^p^ represents the chemical potential of solute *i* in the pure solute.

The chemical reaction for the esterification of AA and PM is represented by Equation (7).

(7) $$\mathrm{AA}+\mathrm{PM}\;⇄{\;\mathrm{PMA}+H}_{2}O$$

This reaction is an acid-catalyzed esterification and is subject to thermodynamic equilibrium. The reaction equilibrium constant based on mole fractions (*K_x_*) is given by Equation (8).

(8) $$K_{x}=\prod x_{i}^{\nu_{i}}=\frac{x_{\mathrm{PMA}}x_{H_{2}O}}{x_{\mathrm{PM}}x_{\mathrm{AA}}}$$

The reaction equilibrium constant based on activities (*K_α_*) is predicted by Equation (9).

(9) $$K_{\alpha }=\prod \alpha_{i}^{\nu_{i}}=\prod {\left(x_{i}\gamma_{i}\right)}^{\nu_{i}}=\frac{x_{\mathrm{PMA}}x_{H_{2}O}}{x_{\mathrm{PM}}x_{\mathrm{AA}}}\frac{\gamma_{\mathrm{PMA}}\gamma_{H_{2}O}}{\gamma_{\mathrm{PM}}\gamma_{\mathrm{AA}}}$$

The calculated activity coefficients for each component corresponding to the experimental mole fraction at equilibrium within the temperature range of 333 to 363 K are listed in Table 3. The calculated activity coefficients for each component corresponding to the experimental mole fraction at equilibrium within the temperature range of 333 to 363 K are listed in Table 2. The activity coefficient of PM ranges from 0.74 to 0.89, which is below 1, indicating strong attractive interactions between PM and other components. This could be attributed to the presence of oxygen atoms and polar hydroxyl groups in PM molecules, which facilitate hydrogen bonding or van der Waals forces with other polar molecules like AA or H_2_O. Conversely, the high activity coefficient of H_2_O ranges from 1.7 to 1.9, indicating strong repulsive interactions between water and the other components, which is consistent with the typical non-ideal behavior observed when water is mixed with non-polar components. The activity coefficients of AA and PMA are from 0.90 to 0.94, and from 1.1 to 1.2, respectively, and being close to 1 suggests that their behavior in the solution approaches an ideal mixture.

The values of *K_x_* and *K_α_* are calculated using Equations (11) and (12). According to the van’t Hoff equation, the relationship between the reaction equilibrium constant and temperature is given by Equation (10).

(10) $$\ln\;K=\frac{-{∆}_{r}H^{\theta }}{RT}+\frac{{∆}_{r}S^{\theta }}{R}$$

**Figure 6:**
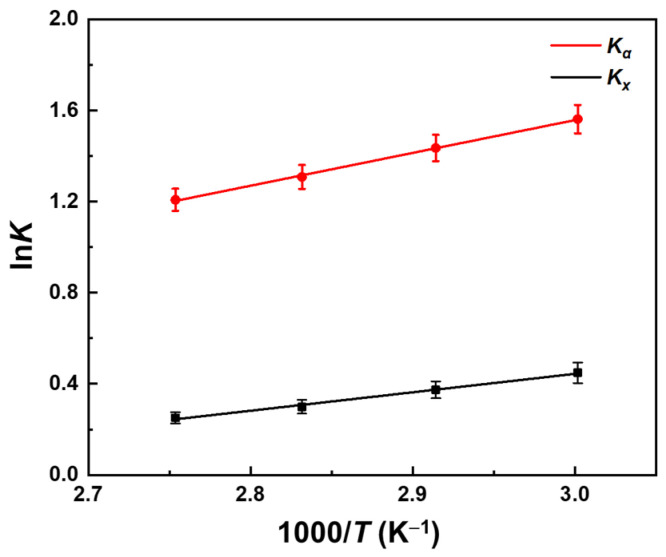
Relationship between ln*K_x_*/ln*K_α_* and 1000/*T*.

A linear fit was performed by the ln of the calculated equilibrium constants *K_x_* and *K_α_* versus the inverse of experimental temperature values. The fitting results are shown in Figure 6, and the calculated values of the standard enthalpy of the reaction (Δ_r_*H^θ^*) and the standard entropy of the reaction (Δ_r_*S^θ^*) are presented in Table 4:

The expressions relating *K_x_* and *K_α_* to temperature (*T*) are given by Equations (11) and (12).

(11) $$\ln\;K_{x}=\frac{809.92\pm 43.58}{T}-(1.99\pm 0.13)$$

(12) $$\ln\;K_{\alpha }=\frac{1440.27\pm 34.67}{T}-(2.76\pm 0.10)$$

The standard Gibbs free energy of the reaction (Δ_r_*G^θ^*) can be calculated by Equation (13).

(13) $${∆}_{r}G^{\theta }={∆}_{r}H^{\theta }-T{∆}_{r}S^{\theta }$$

The standard enthalpy of the reaction was determined to be −11.97 ± 0.29 kJ/mol (*K_α_*) based on activity calculations, while the standard enthalpy based on mole fraction calculations was −6.73 ± 0.36 kJ/mol (*K_x_*). The standard enthalpy of the reaction based on activity calculation considers the intricate interactions between molecules in the solution, thereby enhancing the accuracy of the resulting data. Previous studies have indicated that the relationship between the equilibrium constant of the reaction and temperature is not particularly strong, suggesting a relatively low value for Δ_r_*H* [13,54], which is consistent with our results. These results indicate that the influence of temperature on the initial reaction rate was more significant than its impact on the equilibrium conversion rate, and the reaction is exothermal. A negative reaction entropy value indicates a reduction in the degree of chaos within the system. The standard Gibbs free energy of the reaction is calculated to be −5.12 ± 0.38 kJ/mol using Equation (13), indicating that the reaction is spontaneous; however, the reaction could not take place owing to the very slow reaction rate at a standard state.

### 2.4. Reaction Kinetic Modelling

Both internal and external mass transfer resistances have been eliminated, as shown in Figure 1 and Table 1, and thus, the reaction rate is dependent on the adsorption of the reaction components on the heterogeneous catalyst. The pseudo-homogeneous (PH), Eley–Rideal (ER), and Langmuir–Hinshelwood–Hougen–Watson (LHHW) kinetic models are frequently employed to correlate kinetic data pertaining to esterification reactions.

The PH model is widely applied in esterification systems, where the adsorption and desorption of all components can be neglected [52,55,56,57,58,59]. The PH model assumes that the catalyst swells completely upon contact with a polar solvent and that the cation exchange resin is equivalent to a liquid acid center, treating the entire reaction system as a homogeneous phase. Both the LHHW and ER models are suitable and applicable for multiphase catalytic reactions when the surface reaction is the controlling step. The LHHW model is effective in describing surface reactions between adsorbed molecules, while the ER model is well-suited to describing surface reactions between an adsorbed substance and a free substance in the liquid phase.

The esterification reaction between AA and PM is reversible, and an excess amount of AA is added in order to enhance the conversion of PM. Consequently, the reaction rate is expressed as the consumption rate of PM. The formulations for the PH, ER, and LHHW models are presented in Equations (14)–(16):

(14) $$r_{\mathrm{PM}}=\frac{dC_{\mathrm{PM}}}{dt}=k_{+}(C_{\mathrm{PM}}C_{\mathrm{AA}}-\frac{1}{K_{x}}C_{\mathrm{PMA}}C_{H_{2}O})$$

(15) $$r_{\mathrm{PM}}=\frac{dC_{\mathrm{PM}}}{dt}=\frac{k_{+}(C_{\mathrm{PM}}C_{\mathrm{AA}}-(1/K_{x})C_{\mathrm{PMA}}C_{H_{2}O})}{\left(1+K_{\mathrm{PM}}C_{\mathrm{PM}}+K_{H_{2}O}C_{H_{2}O}\right)}$$

(16) $$r_{\mathrm{PM}}=\frac{dC_{\mathrm{PM}}}{dt}=\frac{k_{+}(C_{\mathrm{PM}}C_{\mathrm{AA}}-(1/K_{x})C_{\mathrm{PMA}}C_{H_{2}O})}{{\left(1+K_{\mathrm{AA}}C_{\mathrm{AA}}+K_{\mathrm{PM}}C_{\mathrm{PM}}+K_{\mathrm{PMA}}C_{\mathrm{PMA}}+K_{H_{2}O}C_{H_{2}O}\right)}^{2}}$$

where *C*_PM_, *C*_AA_, *C*_PMA_, and *C*_H2O_ are the molar concentrations of PM, AA, PMA, and H_2_O, respectively. *K_x_* is the reaction equilibrium constant based on mole fractions, *K_i_* represents the adsorption equilibrium constant of component *i*, and *k_+_* is the rate constant of the forward reaction.

In consideration of the non-ideality of the liquid phase, the activity-based kinetic models are represented by Equations (17)–(19):

(17) $$r_{\mathrm{PM}}=\frac{dC_{\mathrm{PM}}}{dt}=k_{+}(\alpha_{\mathrm{PM}}\alpha_{\mathrm{AA}}-\frac{1}{K_{\alpha }}\alpha_{\mathrm{PMA}}\alpha_{H_{2}O})$$

(18) $$r_{\mathrm{PM}}=\frac{dC_{\mathrm{PM}}}{dt}=\frac{k_{+}(\alpha_{\mathrm{PM}}\alpha_{\mathrm{AA}}-(1/K_{\alpha })\alpha_{\mathrm{PMA}}\alpha_{H_{2}O})}{\left(1+K_{\mathrm{PM}}\alpha_{\mathrm{PM}}+K_{H_{2}O}\alpha_{H_{2}O}\right)}$$

(19) $$r_{\mathrm{PM}}=\frac{dC_{\mathrm{PM}}}{dt}=\frac{k_{+}(\alpha_{\mathrm{PM}}\alpha_{\mathrm{AA}}-(1/K_{\alpha })\alpha_{\mathrm{PMA}}\alpha_{H_{2}O})}{{\left(1+K_{\mathrm{AA}}\alpha_{\mathrm{AA}}+K_{\mathrm{PM}}\alpha_{\mathrm{PM}}+K_{\mathrm{PMA}}\alpha_{\mathrm{PMA}}+K_{H_{2}O}\alpha_{H_{2}O}\right)}^{2}}$$

where α_PM_, α_AA_, α_PMA_, and α_H2O_ represent the activities of PM, AA, PMA, and H_2_O, respectively, and *K_α_* is the reaction equilibrium constant based on activities.

The adsorption equilibrium amount (*q_e_*, mg/g) of each component on the solid acid catalyst is calculated according to Equation (20):

(20) $$q_{e}=\frac{V(C_{0}-C_{e})}{m}$$

where *C*_0_ and *C*_e_ (g/L) represent the mass concentrations of the solution before adsorption and at equilibrium, respectively, *V* (L) is the volume of the solution, and *m* (g) is the mass of the catalyst.

The Langmuir adsorption isotherm model is employed with relevant expression, which is provided by Equation (21):

(21) $$\frac{1}{q_{e}}=\frac{1}{q_{m}K_{i}}\frac{1}{C_{e}}+\frac{1}{q_{m}}$$

where *K_i_* is the adsorption equilibrium constant, *C*_e_ (g/L) is the mass concentration of the solution at adsorption equilibrium, and *q*_m_ (g/g) represents the theoretical maximum adsorption capacity of the solid acid catalyst.

According to the Arrhenius equation, the relationship between *k_+_* and reaction temperature (*T*) is given by Equation (22):

(22) $$k_{+}=k_{0+}\exp(\frac{-Ea_{+}}{RT})$$

where *k*_0+_ is the pre-exponential factor of the reaction, *Ea*_+_ (kJ/mol) is the activation energy of the reaction, and R is the gas constant.

As both the ER model and LHHW model involve the adsorption of components from the liquid phase onto solid acid catalysts, adsorption experiments were conducted as a preliminary step. The Langmuir adsorption isotherm model was employed, and its expression is given by Equation (20). The *q*_m_ of the solid acid catalyst and *K_i_* obtained from the Langmuir model are presented in Table 5.

Table 5 presents the adsorption equilibrium the constants of PM, AA, PMA, and H_2_O on Amberlyst-35 at 303 K. The variation in the adsorption equilibrium constants with temperature follows the van’t Hoff rule. Generally, as the temperature increased by 10 K, the adsorption equilibrium constants decreased 10–30%. Since highly accurate adsorption equilibrium constants are not required for fitting kinetic models, the predicted adsorption equilibrium constants are within a temperature range of 333 to 363 K based on experimental values at 313 K and reports in the literature [53,60], and are presented in Table 6.

The obtained adsorption equilibrium constants exhibit considerable error, rendering them unsuitable for quantitative analysis. They can, however, be employed as qualitative references. Consequently, these parameter values were employed as initial values for the fitting calculations of the kinetic models, rather than being directly utilized as the adsorption equilibrium constant terms in the ER and LHHW models.

**Figure 7:**
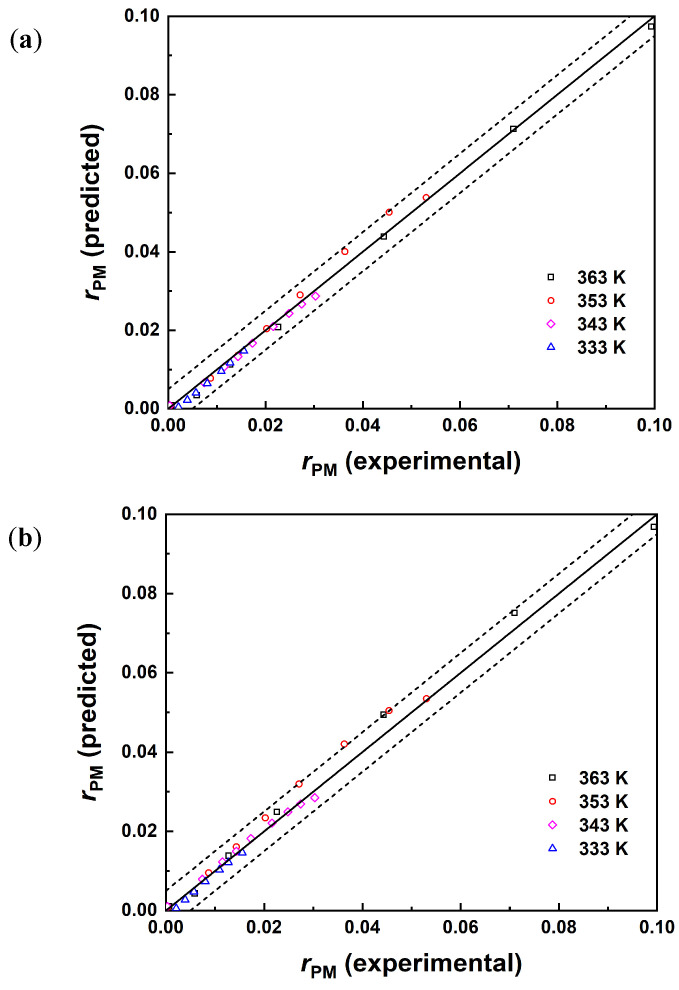

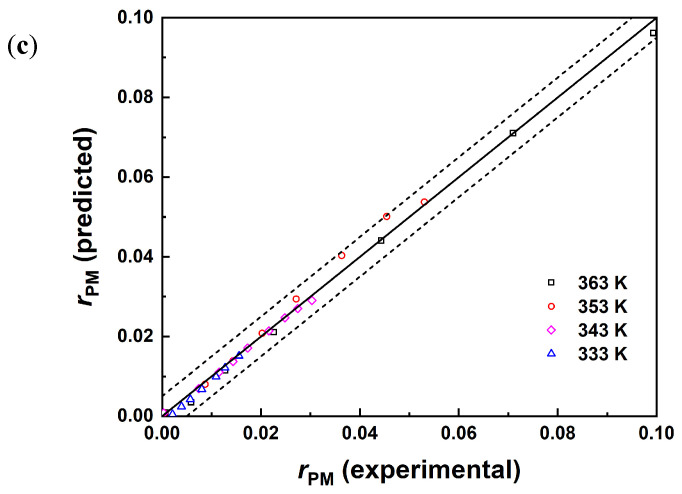
Parity plots for the experimental and predicted rate of reaction for (**a**) PH, (**b**) ER, and (**c**) LHHW.

Python is a high-level programming language that is widely used in scientific computing and data analysis. Due to the non-ideality of the system, the kinetic models were fitted using the activity-based Equations (17)–(19), employing the Python 3.8 programming language. The kinetic parameters and error indicators are presented in Table 7. Although all models exhibit similar values for the root mean square error (RMSE) and coefficient of determination (*R*^2^), the LHHW model had the smallest mean absolute error (MAE). Consequently, among the three models employed, the LHHW model exhibited the most favorable correlation. The parity plot for the experimental and predicted rate of reaction is shown in Figure 7, indicating that the predicted concentration values of the three models are in good agreement with the experimental values.

According to the fitting results of the LHHW model, the apparent activation energy for the esterification reaction between AA and PM catalyzed by Amberlyst-35 is determined to be 62.0 ± 0.2 kJ/mol. This result is in close agreement with the reported value of 60.5 kJ/mol for the forward reaction activation energy by Wang B et al. [14]. The satisfactory correlation between the experimental data and LHHW model suggests that the reaction is governed by surface reactions, which is in accordance with the findings in Section 3.2 regarding the impact of temperature on the esterification reaction. 

According to the LHHW model, PM and AA are independently adsorbed on the catalyst surface (σ), as illustrated in Equations (23) and (24), after which a surface reaction occurs to form PMA, as shown in Equation (25). Finally, produced PMA and H_2_O are desorbed and diffuse into the liquid phase, as shown in Equations (26) and (27).

(23) PM+σ ⇄ PM⋅σ

(24) AA+σ ⇄ AA⋅σ

(25) $$\mathrm{PM}\cdot \sigma +\mathrm{AA}\cdot \sigma \;⇄\;\mathrm{PMA}\cdot \sigma +{\;H}_{2}O\cdot \sigma$$

(26) PMA⋅σ ⇄ PMA +σ

(27) $$H_{2}O\cdot \sigma \;⇄{\;H}_{2}O\;+\sigma$$

## 3. Experimental

### 3.1. Material

Acetic acid (AA, ≥99%), 1-methoxy-2-propanol (PM, ≥99%), and 1-methoxy-2-propyl acetate (PMA, ≥99%) were supplied by Aladdin Biochemical Technology Co., Ltd (Shanghai, China). The chemicals were directly used as received without further purification. The ion-exchange resin Amberlyst-35 was supplied by Shanghai Eastern Rohm & Haas Co., Ltd (Shanghai, China). Before use, the catalyst was placed in a muffle furnace at 110 °C for 8 h to remove moisture and impurities.

### 3.2. Esterification Reaction

The esterification reaction was conducted in a three-necked, round-bottom flask with a volume of 100 mL. A condenser was attached to the top of the flask to prevent the loss of components with anti-backflow suction. The solid acid catalyst and a fixed molar ratio of AA and PM were loaded into the flask. Once the seal was confirmed, the flask was placed in a preheated oil bath with a specific temperature under continuous stirring. The acid-to-ether ratio was within the range of 1:1 to 1:4, and the temperature was within the range of 333 to 363 K. The catalyst Amberlyst-35 was employed in quantities of 5 to 12% in the total mass of AA and PM. Subsequent to the initiation of the reaction, the sample was collected at regular intervals. A precise quantity of 0.5 mL of the liquid sample was taken using a syringe, which was immediately cooled in order to prevent further reaction, and filtered using a needle filter.

In order to determine the adsorption equilibrium constants of the solid acid catalyst with each component, a series of independent experiments were conducted. The catalyst Amberlyst-35 was added to test tubes containing AA, PM, PMA, and H₂O, which were then vigorously stirred at room temperature using a constant temperature oscillator (SD2-100, China) at 800 rpm for 8 h. After adsorption was complete, the supernatant was collected and prepared for analysis.

### 3.3. Product Analysis

The concentrations of AA, PM, and PMA were determined using an Agilent 7890A gas chromatograph (USA) equipped with a flame ionization detector (FID) and an HP-FFAP chromatographic column (30 m × 0.32 mm × 0.25 μm). The analysis was conducted under the following conditions: nitrogen gas with a purity of 0.999 was used as a carrier gas, and the injector and detector temperatures were maintained at 150 °C. The initial column temperature of 60 °C was increased to 90 °C at a rate of 10 °C/min, kept for 1 min, and then rapidly heated to 230 °C at a rate of 30 °C/min. Toluene was used as an internal standard for quantitative analysis.

The conversion of PM (*X*_PM_) and the yield of PMA (*Y*_PMA_) were calculated using Equations (28) and (29), respectively:

(28) $$X_{\mathrm{PM}}=\frac{\mathrm{Mole}\;\mathrm{of}\;\mathrm{PM}\;\mathrm{reacted}}{\mathrm{Mole}\;\mathrm{of}\;\mathrm{PM}\;\mathrm{initially}}$$

(29) $$Y_{\mathrm{PMA}}=\frac{\mathrm{Mole}\;\mathrm{of}\;\mathrm{PMA}\;\mathrm{produced}}{\mathrm{Mole}\;\mathrm{of}\;\mathrm{PM}\;\mathrm{initially}}$$

In all experiments, mass balance was maintained within ±5%. The yield of PMA was accurate within a range of ±2–5%, while the conversion of PM was accurate within ±3–6%.

For each experiment, a data point represented the average of three independent measurements and an error bar indicated the standard deviation. In the direct esterification of PM with AA, the PMA yield versus time exhibited an exponential trend, so the trend line was fitted with the ExpAssoc function, which can clearly visualize the data trend.

The formation rate of PMA (*r*_PMA_) was calculated from the slope of the produced PMA (mol) vs. time (s) and dry catalyst mass (*W*, g) by means of Equation (30):

(30) $$r_{\mathrm{PMA}}(t)=\frac{1}{W}{\left(\frac{dn_{\mathrm{PMA}}}{dt}\right)}_{t}$$

## 4. Conclusions 

The ion-exchange resin Amberlyst-35 was employed as a catalyst for the esterification reaction of PM and AA to form PMA. The impacts of varying reaction parameters, including temperature, catalyst dosage, the initial molar ratio of reactants, and stirring speed on the reaction were investigated. Under optimized reaction conditions with initial reactant PM to AA molar ratio of 1:3 and catalyst loading of 10 wt% for a period of 3 h at 353 K, the equilibrium yield of PMA could reach 78%. A reaction thermodynamic equilibrium model was considered to analyze the reaction’s enthalpy and entropy changes. Furthermore, reaction kinetic models, including PH, ER, and LHHW, were fitted, and the LHHW model demonstrated the most favorable correlation, indicating that the reaction was controlled by surface reactions. The reaction thermodynamic equilibrium and kinetic models serve as a promising foundation to support further efforts to develop economical and environmentally-friendly PMA industrial production routes through the application of ion-exchange resin catalysts.

## Figures and Tables

**Table 1:** Calculated infinite dilution diffusion coefficient (*D*_A_), effective diffusion coefficient (*D*_e_), and Weisz–Prater number (*C*_wp_) at different temperatures.

Temperature (K)	*C*_s_ (mol/cm^3^)	−*r*_obs_ (mol/(s·g))	*D*_A_ (cm^2^/s)	*D*_e_ (cm^2^/s)	*C*_wp_
333.15	1.484 × 10^−2^	2.525 × 10^−6^	2.307 × 10^−5^	2.423 × 10^−6^	0.03
343.15	1.483 × 10^−2^	4.973 × 10^−6^	2.576 × 10^−5^	2.705 × 10^−6^	0.05
353.15	1.482 × 10^−2^	8.726 × 10^−6^	2.841 × 10^−5^	2.983 × 10^−6^	0.08
363.15	1.482 × 10^−2^	1.589 × 10^−5^	3. 990 × 10^−5^	3.254 × 10^−6^	0.13

**Table 2:** Comparison of PMA yield for various catalysts reported in references.

Catalyst	Temperature (K)	Initial Molar Ratio (PM:AA)	Catalyst Loading (wt%)	Yield of PMA (%)	Reference
SO_4_^2−^/TiO_2_	383.15	1:3	10	73	[3]
NKC-9	353.15	1:1	10	46	[14]
Amberlyst-15	353.15	1:3	10	78	[12]
Amberlyst-35	353.15	1:3	10	78	This work
Amberlyst-35	353.15	1:1	10	55	This work

**Table 3:** Mole fractions and evaluated activity coefficients of components in the equilibrium state of the reaction at various temperatures (initial composition of PM:AA = 1:3).

Temperature (K)	PM			AA			PMA			H_2_O		
	*x*_PM_	*γ*_PM_	*α*_PM_	*x*_AA_	*γ*_AA_	*α*_AA_	*x*_PMA_	*γ*_PMA_	*α*_PMA_	*x*_H2O_	*γ*_H2O_	*α*_H2O_
333.15	0.0547	0.8870	0.0485	0.5516	0.9333	0.5148	0.1969	1.187	0.2337	0.1969	1.816	0.3575
343.15	0.0530	0.8433	0.0447	0.5504	0.9239	0.5085	0.1983	1.179	0.2339	0.1983	1.812	0.3593
353.15	0.0502	0.7963	0.0400	0.5493	0.9135	0.5018	0.2002	1.169	0.2340	0.2002	1.800	0.3604
363.15	0.0470	0.7447	0.0350	0.5504	0.9033	0.4972	0.2013	1.152	0.2319	0.2013	1.777	0.3577

**Table 4:** Thermodynamic parameters of the reaction.

Equilibrium Constant	Δ_r_*H^θ^* (kJ/mol)	Δ_r_*S^θ^* (J/(mol·K))	*R* ^2^
*K_x_*	−6.73 ± 0.36	−16.5 ± 1.0	0.9942
*K_α_*	−11.97 ± 0.29	−23.0 ± 0.8	0.9988

**Table 5:** Fitting calculation results of Langmuir adsorption isotherm models at 303 K.

Component	*q*_m_ (g/g)	*K_i_* (L/g)	*R* ^2^
PM	0.4768	0.0271	0.995
AA	0.3582	0.0250	0.833
PMA	2.1921	0.0001	0.926
H_2_O	0.7357	0.0071	0.953

**Table 6:** Predicted values of adsorption equilibrium constants for each component at different temperatures.

Temperature (K)	*K*_PM_ (L/g)	*K*_AA_ (L/g)	*K*_PMA_ (mL/g)	*K*_H2O_ (L/g)
333.15	0.0233	0.0203	0.0590	0.0059
343.15	0.0193	0.0170	0.0550	0.0046
353.15	0.0150	0.0140	0.0510	0.0035
363.15	0.0107	0.0099	0.0470	0.0022

**Table 7:** Kinetic parameters for different models.

Model	*k*_0+_	*Ea*_+_ (kJ/mol)	*K*_PM_	*K*_AA_	*K*_PMA_	*K*_H2O_	*MAE*	*RMSE*	*R* ^2^
PH	2.88 × 10^6^	63.2 ± 0.3	/	/	/	/	1.26 × 10^−3^	1.59 × 10^−3^	0.9964
ER	4.25 × 10^6^	63.3 ± 0.2	0.12	/	/	0.01	1.22 × 10^−3^	1.57 × 10^−3^	0.9947
LHHW	6.84 × 10^6^	62.0 ± 0.2	0.13	0.04	8.2 × 10^−4^	0.15	1.13 × 10^−3^	1.59 × 10^−3^	0.9946

## Data Availability

The data presented in this study are available in this article.
